# Association Between Thionamides and Acute Pancreatitis: A Case–Control Study

**DOI:** 10.1089/thy.2019.0589

**Published:** 2020-11-05

**Authors:** Jia-Yin Guo, Chia-Ling Chang, Ching-Chu Chen

**Affiliations:** ^1^ Department of Medicine, China Medical University, Taichung, Taiwan.; ^2^ Division of Endocrinology and Metabolism, Department of Medicine, China Medical University Hospital, Taichung, Taiwan.; ^3^ Management Office for Health Data, China Medical University Hospital, Taichung, Taiwan.; ^4^ School of Medicine, China Medical University, Taichung, Taiwan.; ^5^ Department of Medicine, School of Chinese Medicine, China Medical University, Taichung, Taiwan.

**Keywords:** thionamides, carbimazole, methimazole, propylthiouracil, pancreatitis, hyperthyroidism

## Abstract

***Background:*** Thionamides have been extensively used to treat patients with hyperthyroidism worldwide. Recent pharmacovigilance studies have revealed a safety signal between carbimazole or methimazole and pancreatitis. The associated risk remains unclear.

***Methods:*** We identified patients with newly diagnosed acute pancreatitis from 2000 to 2013 as the case group from the Taiwan Longitudinal Health Insurance Database 2000, which contains data from 1996 to 2013. Each patient with acute pancreatitis was matched for age, sex, comorbidities, and cancer with four controls through propensity score matching. A total of 52 patients without matched controls were excluded. Sensitivity analyses including the 52 excluded patients were performed using a matching ratio of 1:2. Odds ratios (ORs) along with 95% confidence intervals (CIs) for the association were estimated using multivariate logistic regression.

***Results:*** We included 9204 and 36,816 patients in the case and control groups, respectively. The proportions of patients who had used thionamides, carbimazole, methimazole, and propylthiouracil were similar in these two groups. In addition, the adjusted OR (CI) for the association of acute pancreatitis with thionamides was 1.03 (0.86–1.24), with carbimazole it was 0.90 (0.63–1.30), with methimazole it was 1.05 (0.84–1.31), and with propylthiouracil it was 1.00 (0.74–1.34). The sensitivity analysis results were unchanged.

***Conclusions:*** We were unable to demonstrate an association between acute pancreatitis and usage of thionamides.

## Introduction

Thionamides have been used to treat patients with hyperthyroidism for many years. On January 4, 2019, the Pharmacovigilance Risk Assessment Committee of the European Medicines Agency published a recommendation regarding the safety signals of carbimazole and methimazole (1). The committee recommended that carbimazole or methimazole should be discontinued immediately if patients develop acute pancreatitis during treatment, and that for patients with a history of acute pancreatitis, clinicians should avoid re-exposing patients to carbimazole or methimazole after a previous administration of one of these drugs. The Medicines and Health care Products Regulatory Agency of the United Kingdom followed these recommendations and published the same safety signal on February 18, 2019 (2). However, the associated risk remains clinically unclear.

The aim of this retrospective case–control study was to evaluate the associations of thionamides in general and carbimazole, methimazole, and propylthiouracil in particular with acute pancreatitis using Taiwan's Longitudinal Health Insurance Database 2000 (LHID2000). This study was approved by the Research Ethics Committee of China Medical University and Hospital (CMUH-104-REC2-115-R3).

## Methods

### Study population

The LHID2000 contains all claims data for 1 million insured subjects from 1996 to 2013, who were randomly selected from Taiwan's National Health Insurance Research Database. International Classification of Diseases, Ninth Revision, Clinical Modification (ICD-9-CM) codes are used to define disease diagnoses from outpatient and inpatient data in the LHID2000. The ICD-9-CM codes used in this study are presented in Supplementary Table S1.

We identified patients with new diagnoses of acute pancreatitis—defined as ≥2 outpatient visit diagnoses or ≥1 diagnosis during hospitalization or an emergency visit—as the case group. The exclusion criteria included the following: index date not within the study period (2000–2013), age younger than 20 years, age older than 100 years, and missing sex or age data. The diagnosis date was defined as the index date. We used the same exclusion criteria to identify patients without a history of acute pancreatitis as the control group; these controls were matched for age, sex, comorbidities (including alcoholic liver disease, gallbladder stone, hyperlipidemia, and type 2 diabetes mellitus), and cancer with the case group through propensity score matching at a ratio of 1:4. A total of 52 patients without matched controls were excluded. Sensitivity analyses including the 52 excluded patients were performed at a matching ratio of 1:2 to clarify the effect of excluding the 52 unmatched patients on the association. The status of antithyroid drug use was categorized as never use and ever use.

### Statistical analyses

Chi-square tests were used to compare categorical variables. Odds ratios (ORs) along with 95% confidence intervals (CIs) for the association were estimated using multivariate logistic regression. The ORs were adjusted for age, sex, alcoholic liver disease, gallbladder stone, and cancer. All statistical analyses were performed using STATA/SE version 14.0 (STATA Corp., College Station, TX). Results with a two-sided *p*-value of <0.05 were considered significant.

## Results

**FIG. 1.**
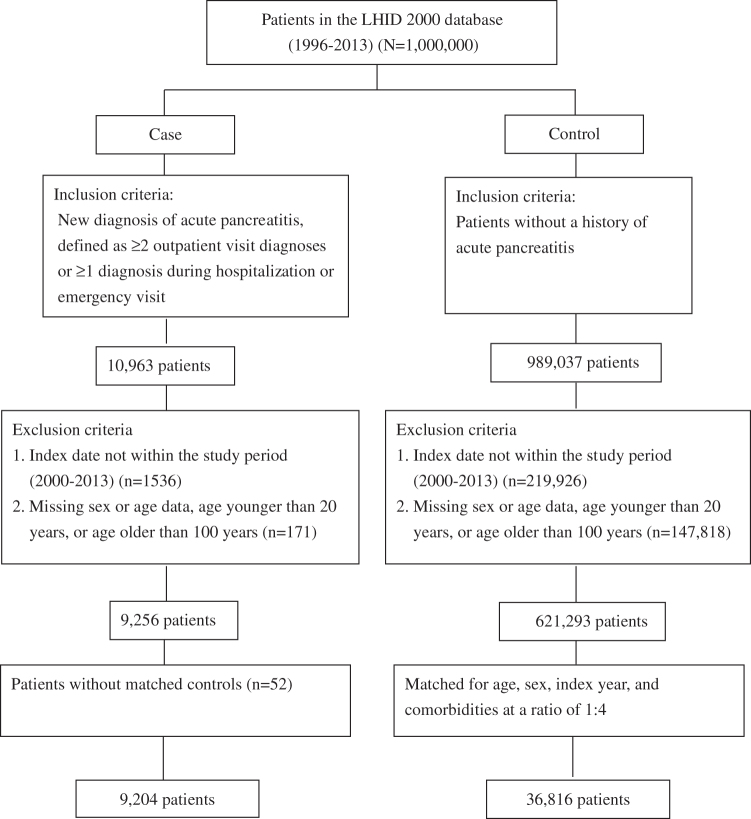
Flowchart of included patients. LHID, Longitudinal Health Insurance Database.

As shown in Figure 1, from the total of 1 million patients in the LHID2000, 10,963 patients with newly diagnosed acute pancreatitis were included in the case group. We excluded 1536 patients for whom the date of diagnosis was not within the study period; 171 patients without sex or age data, aged <20 years, or aged >100 years; and 52 patients without matched controls (including 3 patients who had been prescribed thionamides). Finally, a total of 9204 patients were included in the case group. For the control group, we identified 989,037 patients without a history of acute pancreatitis. From these patients, 234,744 were excluded using the same exclusion criteria as the case group. Finally, the control group comprised 36,816 patients who were matched with the case group for age, sex, index year, and comorbidities at a ratio of 1:4.

As presented in Table 1, the proportions of patients who had ever used thionamides (1.59% vs. 1.58%), carbimazole (0.39% vs. 0.44%), methimazole (1.10% vs. 1.07%), and propylthiouracil (0.61% vs. 0.61%) were similar in the case and control groups. Patients with acute pancreatitis were younger than those without acute pancreatitis. The proportion of patients with alcoholic liver disease was higher in the case group than in the control group; however, the proportions of patients with gallbladder stone and cancer were lower in the case group than in the control group.

**Table 1.** Characteristics of Patients

	Acute pancreatitis		p
	Yes (case) (*n* = 9204),* n *(%)	No (control) (*n* = 36,816),* n *(%)	
Thionamides			0.970
Never	9058 (98.41)	36,234 (98.42)	
Ever	146 (1.59)	582 (1.58)	
Carbimazole			0.539
Never	9168 (99.61)	36,655 (99.56)	
Ever	36 (0.39)	161 (0.44)	
Methimazole			0.822
Never	9103 (98.90)	36,422 (98.93)	
Ever	101 (1.10)	394 (1.07)	
Propylthiouracil			0.976
Never	9148 (99.39)	36,591 (99.39)	
Ever	56 (0.61)	225 (0.61)	
Sex			0.744
Women	3516 (38.20)	14,132 (38.39)	
Men	5688 (61.80)	22,684 (61.61)	
Age			<0.001
<40	2382 (25.88)	7639 (20.75)	
40–65	4066 (44.18)	16,999 (46.17)	
≥65	2756 (29.94)	12,178 (33.08)	
Comorbidity			
Alcoholic liver disease			<0.001
Without	8198 (89.07)	33,612 (91.30)	
With	1006 (10.93)	3204 (8.70)	
Gallbladder stone			<0.001
Without	6768 (73.53)	26,252 (71.31)	
With	2436 (26.47)	10,564 (28.69)	
Hyperlipidemia			0.904
Without	6288 (68.32)	25,128 (68.25)	
With	2916 (31.68)	11,688 (31.75)	
Type 2 diabetes mellitus			0.256
Without	6878 (74.73)	27,723 (75.30)	
With	2326 (25.27)	9093 (24.70)	
Cancer			<0.001
Without	8671 (94.21)	34,310 (93.19)	
With	533 (5.79)	2506 (6.81)	

As indicated in Table 2, the adjusted OR (CI) for acute pancreatitis was 1.03 (0.86–1.24) for thionamides, 0.90 (0.63–1.30) for carbimazole, 1.05 (0.84–1.31) for methimazole, and 1.00 (0.74–1.34) for propylthiouracil. Sensitivity analyses conducted using a matching ratio of 1:2 (9256 patients vs. 18,512 patients; Supplementary Table S2) revealed that the risks of acute pancreatitis remained unchanged in users of all thionamides (1.10 [CI: 0.90–1.35]), carbimazole users (1.00 [CI: 0.67–1.49]), methimazole users (1.08 [CI: 0.85–1.37]), and propylthiouracil users (1.16 [CI: 0.83–1.61]; Supplementary Table S3).

**Table 2.** Association Between Antithyroid Drugs and Acute Pancreatitis

	Thionamides		Carbimazole		Methimazole		Propylthiouracil	
	Adjusted OR (CI)	p	Adjusted OR (CI)	p	Adjusted OR (CI)	p	Adjusted OR (CI)	p
Drug								
Never	Ref.		Ref.		Ref.		Ref.	
Ever	1.03 (0.86–1.24)	0.751	0.90 (0.63–1.30)	0.581	1.05 (0.84–1.31)	0.655	1.00 (0.74–1.34)	0.994
Sex								
Women	Ref.		Ref.		Ref.		Ref.	
Men	0.96 (0.91–1.01)	0.098	0.96 (0.91–1.01)	0.089	0.96 (0.91–1.01)	0.097	0.96 (0.91–1.01)	0.094
Age (years)								
<40	Ref.		Ref.		Ref.		Ref.	
40–65	0.76 (0.72–0.81)	<0.001	0.76 (0.72–0.81)	<0.001	0.76 (0.72–0.81)	<0.001	0.76 (0.72–0.81)	<0.001
≥65	0.75 (0.70–0.80)	<0.001	0.75 (0.70–0.80)	<0.001	0.75 (0.70–0.80)	<0.001	0.75 (0.70–0.80)	<0.001
Comorbidity								
Alcoholic liver disease								
Without	Ref.		Ref.		Ref.		Ref.	
With	1.31 (1.21–1.41)	<0.001	1.31 (1.21–1.41)	<0.001	1.31 (1.21–1.41)	<0.001	1.31 (1.21–1.41)	<0.001
Gallbladder stone								
Without	Ref.		Ref.		Ref.		Ref.	
With	0.96 (0.91–1.02)	0.195	0.97 (0.91–1.02)	0.198	0.96 (0.91–1.02)	0.195	0.96 (0.91–1.02)	0.197
Cancer								
Without	Ref.		Ref.		Ref.		Ref.	
With	0.89 (0.81–0.99)	0.026	0.89 (0.81–0.99)	0.026	0.89 (0.81–0.99)	0.026	0.89 (0.81–0.99)	0.026

Adjusted for age, sex, alcoholic liver disease, gallbladder stone, and cancer.

CI, 95% confidence interval; OR, odds ratio.

## Discussion

This study demonstrates no significant association between usage of thionamides (methimazole, carbimazole, and propylthiouracil) and the development of acute pancreatitis. However, previous studies have reported that acute pancreatitis was associated with methimazole or carbimazole use in nine patients (3–11). Propylthiouracil is the only thionamide without a case report, suggesting an association with the development of acute pancreatitis. As presented in Table 3, the onset of acute pancreatitis symptoms after methimazole or carbimazole exposure varied from 4 to 90 days. All symptoms and laboratory abnormalities resolved after the drug were withdrawn. The recurrent rate of acute pancreatitis after thionamide withdrawal was unknown. However, acute pancreatitis recurred in five of the five patients (four methimazole users and one carbimazole user) who were rechallenged with the original thionamide (3,4,8–10). The interval between rechallenge and the recurrence of acute pancreatitis ranged from 3 hours to 5 days. However, none of the authors mentioned whether these five patients ever experienced acute pancreatitis previously, before ever being exposed to thionamides, or ultimately developed chronic pancreatitis (Supplementary Table S4). Some case reports did not exclude viral infections (8,10) or autoimmune disease (3,8,10) as a potential etiology of acute pancreatitis. Potassium iodide (one patient), propylthiouracil (two patients), and radioactive iodine (four patients) were administrated for the management of hyperthyroidism after acute pancreatitis resolved.

**Table 3.** Case Reports of Methimazole- or Carbimazole-Induced Acute Pancreatitis

Authors	Reporting year	Ethnicity	Age	Sex	Diagnosis	Time of symptom onset	Drug	Dose (mg)	Management
Taguchi *et al.* (3)	1999	Japanese	66	W	Graves' disease	21 days 3 hours after 2nd dose	MMI	30 10^a^	PTU
Marazuela *et al.* (4)	2002	Spanish	33	W	Graves' disease	30 days 1 day	Carbimazole	45 10^a^	RAI
Su *et al.* (5)	2008	Chinese	19	W	Graves' disease	75 days	MMI	10	RAI
Chng *et al.* (6)	2011	Asian	70	W	Graves' disease	14 days	Carbimazole	30	RAI
Abraham *et al.* (7)	2012	Caucasian	80	W	Unspecified	90 days	MMI	10	Unspecified
Yang *et al.* (8)	2012	Chinese	18	W	Graves' disease	4 days Within 1 day	MMI	20 10^b^ 10^b^ 10^b^	PTU
Jung *et al.* (9)	2014	Korean	51	M	Graves' disease	14 days 5 hours	MMI	20 10^a^	Unspecified
Agito *et al.* (10)	2015	Caucasian	51	W	Toxic MNG	21 days 5 days	MMI	10 10^a^	RAI
Kikuchi *et al.* (11)	2018	Japanese	76	W	Graves' disease	19 days	MMI	10	Potassium iodide

There is no report of recurrent rate of acute pancreatitis in patients who were not re-exposed to thionamides.

^a^ Rechallenged.

^b^ Rechallenged three times at different times by three physicians.

M, men; MMI, methimazole; MNG, multinodular goiter; PTU, propylthiouracil; RAI, radioactive iodine; W, women.

The results of the present study are consistent with those of a previous study that reported no association between methimazole and the development of acute pancreatitis (12). Moreover, an animal study revealed that methimazole reduced the severity of cerulean-induced acute pancreatitis in rats (13). An additional study reported that propylthiouracil attenuated pancreatic activity to reduce the mortality rate of hemorrhagic pancreatitis in dogs (14).

The strengths of this study include its large real-world sample, which compensates for the absence of a randomized-controlled study. Nevertheless, despite this strength, some limitations should be acknowledged. First, the exclusion of 52 unmatched patients may have compromised the results; however, sensitivity analyses reinforced the findings. Second, a causal relationship cannot be determined using insurance claims data. Third, although the case and control groups were matched for age, sex, and some comorbidities, potentially important confounders may have been unavailable for adjustment.

In conclusion, we were unable to demonstrate an association between the development of acute pancreatitis and use of thionamides.

## Supplementary Material

Supplemental data

Supplemental data

Supplemental data
