# Supplementary material for: Association Between Thionamides and Acute Pancreatitis: A Case–Control Study
Source: Thyroid. 2020 Nov 5;30(11):1574–8. doi: 10.1089/thy.2019.0589 (PMC7692926; doi:10.1089/thy.2019.0589)
Supplement: Supplemental data [file Supp_TableS1.pdf]

Supplementary Data

Supplementary Table S1. ICD-9-CM and ATC Codes

ICD-9-CM code	
Acute pancreatitis	577.0
Alcoholic liver disease	571.0, 571.1, 571.2, 571.3
Gallbladder stone	574.00, 574.01, 574.10, 574.11, 574.20, 574.21, 574.50, 574.90
Hyperlipidemia	272.1, 272.2
Type 2 diabetes mellitus	250.00, 250.02, 250.10,250.12,250.20,250.22, 250.30, 250.32, 250.40, 250.42, 250.50, 250.52, 250.60, 250.62, 250.70, 250.72, 250.80, 250.82, 250.90, 250.92
Cancer	140–208 with catastrophic card
ATC code	
Carbimazole	H03BB01
Methimazole	H03BB02
Propylthiouracil	H03BA02

ATC, Anatomical Therapeutic Chemical Classification System; ICD-9-CM, International Classification of Diseases, Ninth Revision, Clinical Modification.
