# Supplementary material for: Association Between Thionamides and Acute Pancreatitis: A Case–Control Study
Source: Thyroid. 2020 Nov 5;30(11):1574–8. doi: 10.1089/thy.2019.0589 (PMC7692926; doi:10.1089/thy.2019.0589)
Supplement: Supplemental data [file Supp_TableS2.pdf]

Supplementary Table S2. Characteristics of Patients

	Acute pancreatitis		
	Yes (case) (n = *9,256),* n *(%)*	*No (control)* (n = *18,512),* n *(%)*	p
Thionamides			0.447
Never	9107 (98.39)	18236 (98.51)	
Ever	149 (1.61)	276 (1.49)	
Carbimazole			0.946
Never	9220 (99.61)	18439 (99.61)	
Ever	36 (0.39)	73 (0.39)	
Methimazole			0.622
Never	9152 (98.88)	18316 (98.94)	
Ever	104 (1.12)	196 (1.06)	
Propylthiouracil			0.362
Never	9199 (99.38)	18414 (99.47)	
Ever	57 (0.62)	98 (0.53)	
Sex			0.367
Women	3528 (38.12)	6953 (37.56)	
Men	5728 (61.88)	11559 (62.44)	
Age			<0.001
<40	2389 (25.81)	3834 (20.71)	
40–65	4098 (44.27)	8700 (47.00)	
≥65	2769 (29.92)	5978 (32.29)	
Comorbidity			
Alcoholic liver disease			<0.001
Without	8198 (88.57)	16753 (90.50)	
With	1058 (11.43)	1759 (9.50)	
Gallbladder stone			0.001
Without	6768 (73.12)	13179 (71.19)	
With	2488 (26.88)	5333 (28.81)	
Hyperlipidemia			0.949
Without	6299 (68.05)	12605 (68.09)	
With	2957 (31.95)	5907 (31.91)	
Type 2 diabetes mellitus			0.333
Without	6901 (74.56)	13901 (75.09)	
With	2355 (25.44)	4611 (24.91)	
Cancer			0.026
Without	8721 (94.22)	17315 (93.53)	
With	535 (5.78)	1197 (6.47)	
