# Supplementary material for: Association Between Thionamides and Acute Pancreatitis: A Case–Control Study
Source: Thyroid. 2020 Nov 5;30(11):1574–8. doi: 10.1089/thy.2019.0589 (PMC7692926; doi:10.1089/thy.2019.0589)
Supplement: Supplemental data [file Supp_TableS3-S4.pdf]

Supplementary Table S3. Association Between Antithyroid Drugs and Acute Pancreatitis

	Thionamides		*Carbimazole*		*Methimazole*		*Propylthiouracil*	
	Adjusted OR (CI)	p	*Adjusted OR (CI)*	p	*Adjusted OR (CI)*	p	*Adjusted OR (CI)*	p
Drug								
Never	Ref.		Ref.		Ref.		Ref.	
Ever	1.10 (0.90–1.35)	0.346	1.00 (0.67–1.49)	0.988	1.08 (0.85–1.37)	0.533	1.16 (0.83–1.61)	0.383
Sex								
Women	Ref.		Ref.		Ref.		Ref.	
Men	0.93 (0.88–0.98)	0.098	0.93 (0.88–0.98)	0.006	0.93 (0.88–0.98)	0.006	0.93 (0.88–0.98)	0.007
Age (years)								
<40	Ref.		Ref.		Ref.		Ref.	
40–65	0.75 (0.71–0.80)	<0.001	0.75 (0.71–0.80)	<0.001	0.75 (0.71–0.80)	<0.001	0.75 (0.71–0.80)	<0.001
≥65	0.76 (0.70–0.81)	<0.001	0.76 (0.71–0.81)	<0.001	0.76 (0.71–0.81)	<0.001	0.76 (0.71–0.81)	<0.001
Comorbidity								
Alcoholic liver disease								
Without	Ref.		Ref.		Ref.		Ref.	
With	1.26 (1.16–1.36)	<0.001	1.25 (1.16–1.36)	<0.001	1.26 (1.16–1.36)	<0.001	1.25 (1.15–1.36)	<0.001
Gallbladder stone								
Without	Ref.		Ref.		Ref.		Ref.	
With	0.96 (0.90–1.02)	0.195	0.96 (0.91–1.02)	0.173	0.96 (0.91–1.02)	0.171	0.96 (0.91–1.02)	0.17
Cancer								
Without	Ref.		Ref.		Ref.		Ref.	
With	0.94 (0.85–1.05)	0.026	0.94 (0.85–1.05)	0.288	0.94 (0.85–1.05)	0.288	0.94 (0.85–1.05)	0.289

Adjusted for age, sex, alcoholic liver disease, gallbladder stone, and cancer.
CI, 95% confidence interval; OR, odds ratio.

Supplementary Table S4. Summary of Five Rechallenged Patients

	Taguchi et al. (3)	*Marazuela* et al. (4)	*Yang* et al. (8)	*Jung* et al. (9)	*Agito* et al. (10)
Ever experienced acute pancreatitis previously?	Normal amylase/ lipase before methimazole use	Not mentioned	Not mentioned	Not mentioned	Not mentioned
Before ever being exposed to thionamides?	Unspecified	Unspecified	Yes, propylthiouracil	Unspecified	Unspecified
Chronic pancreatitis developed?	Not mentioned	Not mentioned	Not mentioned	Not mentioned	Not mentioned
Alcohol drinking	None	None	Not mentioned	None	None
Other medications	Not mentioned	None	Not mentioned	Bisoprolol, aspirin	Not mentioned
Hypertriglyceridemia	None	None	None	None	None
Abnormalities in pancreas or hepatobiliary system	None	None	None	None	None
Virus infection associated	None	None	Not mentioned	None	Not mentioned
Autoimmune disease	Not mentioned	Normal IgG, IgA, IgM, IgE levels	Not mentioned	Normal IgG and IgG4 levels	Not mentioned
